# A Rare Primary Osteogenic Sarcoma of the Prostate and Bladder

**DOI:** 10.7759/cureus.15689

**Published:** 2021-06-16

**Authors:** Jesse Rockmore, Gregory McIntosh, John C Pui, Safi Mohammaed, Robert Elgin

**Affiliations:** 1 Urology, Mclaren Macomb/Michigan State University College of Osteopathic Medicine, Clinton Township, USA; 2 Department of Urology, Michigan Institute of Urology, Macomb, USA; 3 Pathology, Beaumont Health, Farmington Hills, USA; 4 Urology, Riverside Health System, Kankakee, USA

**Keywords:** bladder cancer, prostate cancer, osteosarcoma, endourology, cystoprostatectomy

## Abstract

Mesenchymal tumors of the genitourinary tract account for 5% percent of bladder malignancies and there are currently 35 documented cases of osteosarcoma type. Concomitant involvement of the prostate in mesenchymal genitourinary malignancies is even rarer. Herein we describe a case of a 72-year-old male with a history of radiation for prostate cancer who develops hematuria. A hematuria evaluation revealed osteosarcoma of the bladder and prostate. He underwent radical cystoprostatectomy with ileal conduit and adjuvant chemotherapy. His disease progressed despite treatment and he elected palliative care 10 months after initial resection. This case reviews a rare histological variant of genitourinary malignancy.

## Introduction

Of extraskeletal osteosarcomas, those involving the genitourinary tract are rare and account for 0.04% and to date, there have been 35 documented cases [1]. The tumor presents with hematuria and has a 4:1 male to female predominance. Associated risks for osteosarcoma of the bladder include prior radiation therapy and a history of urinary schistosomiasis [2]. Previous reviews reveal an average age of onset of 62 years [3]. Mesenchymal neoplasms of the prostate are rare with few documented cases [4]. Mesenchymal neoplasms of the bladder account for 5% of bladder tumors [5]. 

Macroscopically, the tumor is characterized as large, polyploid, and infiltrative. When involving the bladder, the tumor often arises from the trigone [2]. Microscopically, it is characterized by arising from soft tissue not attached to bone or periosteum, with a uniform sarcomatous pattern, with osteoid or cartilage matrix [6]. Differential diagnosis includes sarcomatoid urothelial carcinoma and urothelial carcinoma with osseous metaplasia [2]. Histological staining for osteosarcoma is negative for pan-cytokeratin 7 and 20, epithelial membrane antigen, smooth muscle actin, desmin, CD34, and CD68. It strongly expresses vimentin and p53 [1]. Osteoid elements are present with mature lamellar structures, keratin, and desmosomes [2].

The prognosis for osteosarcoma is poor in comparison to other differential diagnoses with demise typically occurring within six months. A series examining 25 cases of vesical osteosarcoma found that 22 of the 25 were dead at six months [2]. Local recurrence is common (45%), as well as, distant metastases (65%), with the predominant metastases being pulmonary (81%) [6]. Treatment includes Bacille Calmette-Guerin for superficial malignancy and a combination of radical cystectomy, chemotherapy, and radiation for invasive tumors. Contributing factors to morality include continued local invasion, urinary obstruction, uremia, infection, and pulmonary metastasis [1]. Rare anecdotal cases report survival of 36-51 months with a combination of partial or radical cystectomy with chemotherapy [7].

## Case presentation

A 72-year-old African American man with a history of prostate cancer treated 12 years prior with external beam radiation and brachytherapy presented with hematuria. His prostate-specific antigen (PSA) had been followed and found to be <0.01 without evidence of biochemical recurrence. Office cystoscopy revealed what was thought to be bladder calculi. Rigid cystoscopy revealed an obstructing calcified bladder mass that was resected. Pathology confirmed at the Cleveland Clinic revealed osteosarcoma of the bladder.

Local recurrence after initial negative metastatic workup occurred two months after initial resection and additional transurethral resection was completed. The patient subsequently underwent radical cystoprostatectomy, ileal conduit, colon resection with colostomy due to adherent tumor to the rectum three months after initial resection.

**Figure 1:**
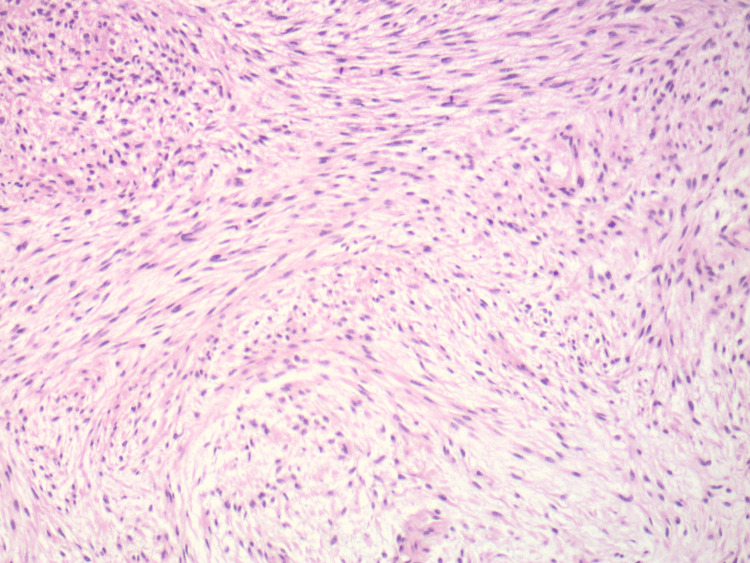
Portions of the tumor show atypical spindle-shaped cells arranged in sweeping fascicles, similar to that seen in fibrosarcoma (H and E, original magnification 100X)

**Figure 2:**
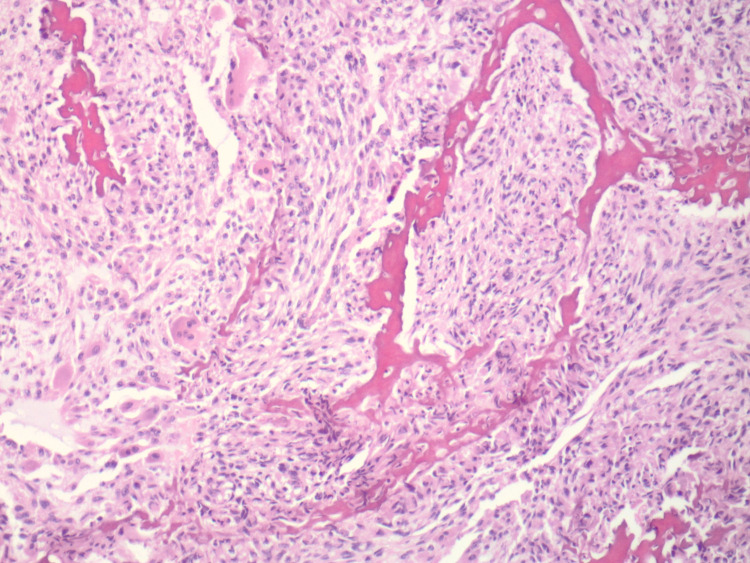
Other portions of the tumor show irregular osteoid formation intimately admixed amongst the atypical spindle-shaped cells (H and E, original magnification 100X)

**Figure 3:**
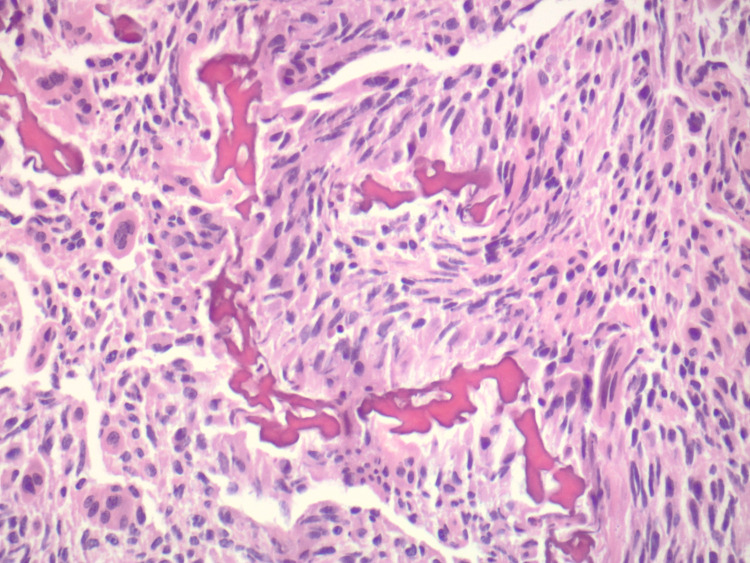
Higher power view of irregular osteoid within atypical cells, with associated osteoclast-like giant cells (H and E, original magnification 200X)

**Figure 4:**
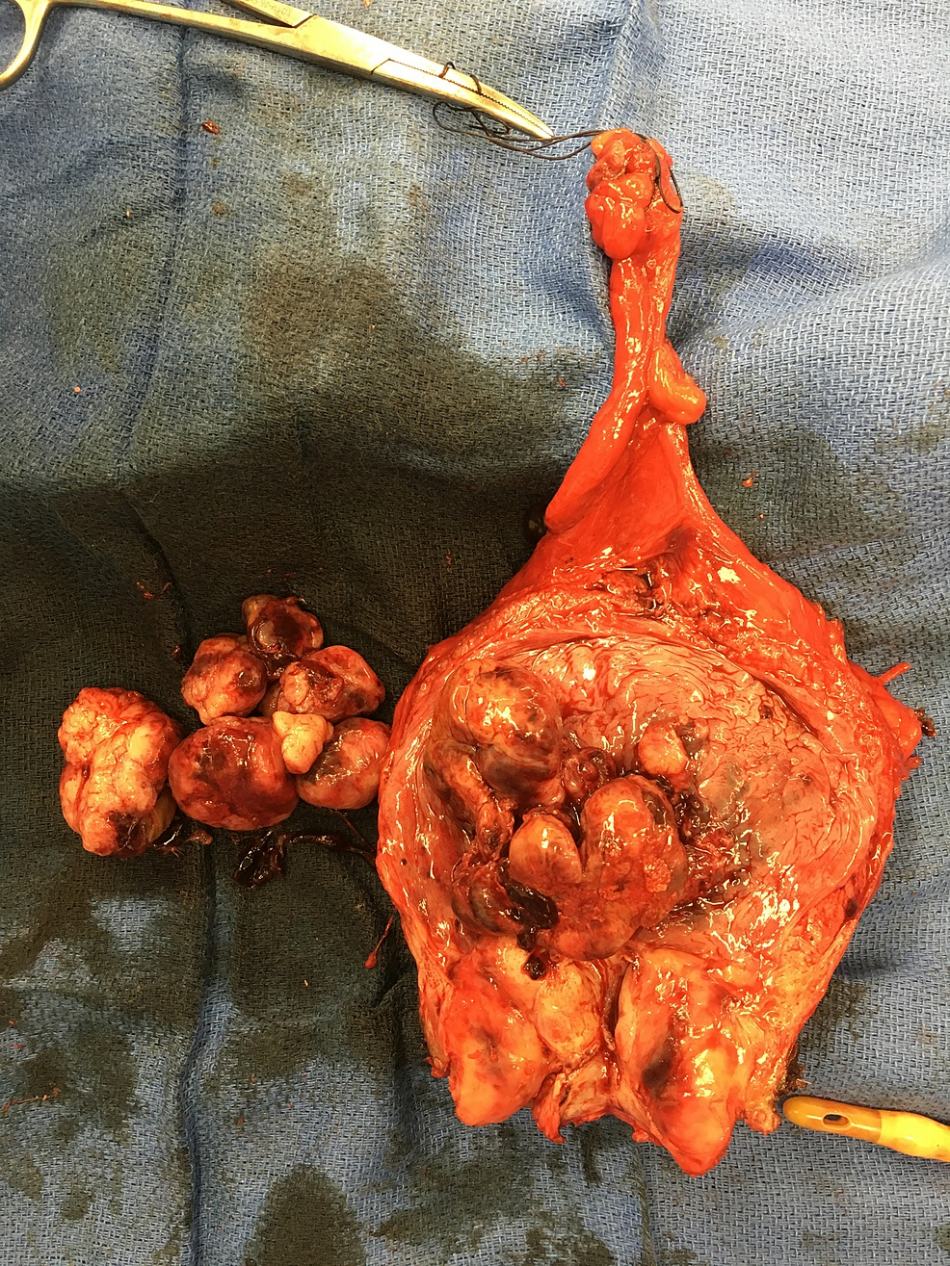
Gross specimen, bladder, prostate, lymph nodes (magnification 0x)

Pathology revealed osteosarcoma replacement to the prostate with extension into the bladder [Figure 1-3], and a single node positive for osteosarcoma. In addition, the gross specimen is seen with a tumor arising from the trigone [Figure 4].

 

He experienced distant metastatic disease to the liver and bone three months following the cystoprostatectomy despite adjuvant chemotherapy. His prognosis was deemed poor and he chose palliative care 10 months after initial resection.

## Discussion

Radiation-induced secondary malignancies are of concern in patients with a history of abdominopelvic malignancies treated with radiation. Cahan et al. developed criteria for determining if a secondary malignancy is radiation-induced: second malignancy occurring within the field of radiation, the histology differs from the primary histology and an incubation period of at least five years [8]. 

Development of sarcomas after prostate radiation is rare. A Surveillance, Epidemiology and End Result Database (SEER) analysis of men who had radiation therapy for prostate cancer showed that 38 of 51,584 developed sarcomas [9]. The relative risk of sarcoma development after radiation for prostate cancer was 1.21 compared to 1.02 of those treated surgically. Sarcoma is a class that includes types other than osteosarcoma, including leiomyosarcoma, fibrosarcoma, rhabdomyosarcoma, and malignant fibrous histiocytoma. Cha et al. found of radiation-induced sarcomas, survival at one, three, and five years if the sarcoma was resectable was 78%, 58%, and 41%, respectively, and a positive surgical margin after resection was associated with 27% five-year survival [10]. This data is not specific to osteosarcoma which highlights the need for more reportable cases of genitourinary osteosarcomas to provide outcome data. 

Furthermore, while our case predominately examines a genitourinary radiation-induced secondary malignancy in a male patient, both genders are at risk. A cohort of men and women undergoing abdominopelvic radiation for primary colorectal or genitourinary cancers were found to have an incidence of 0.3% developing sarcomas after radiation [11]. In addition, age has been examined as a risk factor for radiation-induced secondary malignancy. Recent studies showed an excess risk of secondary malignancy due to radiation sensitivity beyond the age of 45 [12]; a particular consideration for urologists in the treatment of prostate cancer. More analysis is needed to identify prostate cancer patients at increased risk of developing secondary radiation-induced malignancies, and during counseling on treatment modality for prostate cancer, the risk for secondary malignancy should be discussed. 

Our case demonstrates variant histology associated with a rare sequela of radiation therapy. In addition, our institution had yet to encounter this histological variant. Outside evaluation with a national cancer center was necessary to confirm the diagnosis. Osteosarcoma of the prostate and bladder is rare. In this case, the disease progressed despite surgical and oncological treatment. Prostate cancer is a common disease treated with radiation. Rare secondary malignancies from prior radiation reveal a long-term sequela of radiation therapy. Our case highlights a need to counsel patients on the long-term potential adverse effects of radiation therapy for prostate cancer. Proper diagnosis histologically is imperative when confronted with variant pathology. Osteosarcoma portends a worse prognosis when compared to differential diagnoses of sarcomatoid urothelial carcinoma and urothelial carcinoma with osseous metaplasia. A multi-center, multi-disciplinary approach to the treatment of variant histology is essential for proper diagnosis and treatment.

## Conclusions

Variant histology in genitourinary malignancies is at times rare and underreported. Proper referral to specialty pathology centers is necessary for accurate diagnosis of variant histologies. Treatment of variant histologies requires a multi-disciplinary approach to reduce morbidity and mortality. 

## References

[1] Siddappa JK, Singla S, Jain A, Kumar A (2012). A rare case of primary osteosarcoma of urinary bladder. J Clin Imaging Sci.

[2] Ghalayini IF, Bani-Hani IH, Almasri NM (2001). Osteosarcoma of the urinary bladder occurring simultaneously with prostate and bowel carcinomas: report of a case and review of the literature. Arch Pathol Lab Med.

[3] Young RH, Rosenberg AE (1987). Osteosarcoma of the urinary bladder. Report of a case and review of the literature. Cancer.

[4] Nishiyama T, Ikarashi T, Terunuma M, Ishizaki S (2001). Osteogenic sarcoma of the prostate. Int J Urol.

[5] Wong-You-Cheong JJ, Woodward PJ, Manning MA, Sesterhenn IA (2006). From the Archives of the AFIP: neoplasms of the urinary bladder: radiologic-pathologic correlation. Radiographics.

[6] Sadhvani BP, Tourani VK, Rao VN (2014). Osteosarcoma of urinary bladder-a rare but distinct clinicopathological entity: a case report. Research and reviews: journal of medical and health sciences.

[7] Papandreou C, Skopelitou A, Kappes G, Daouaher H (2010). Primary osteosarcoma of the urinary bladder treated with external radiotherapy in a patient with a history of transitional cell carcinoma: a case report. J Med Case Rep.

[8] Cahan WG, Woodard HQ, Higinbotham NL, Stewart FW, Coley BL (1998). Sarcoma arising in irradiated bone: report of eleven cases. 1948. Cancer.

[9] Wakabayashi K, Konishi K, Komatsu T (2019). Radiation-induced sarcoma after radiation therapy for prostate adenocarcinoma. IJU Case Rep.

[10] Cha C, Antonescu CR, Quan ML, Maru S, Brennan MF (2004). Long-term results with resection of radiation-induced soft tissue sarcomas. Ann Surg.

[11] Hird AE, Magee DE, Matta R (2020). Assessment of secondary sarcomas among patients with cancer of the abdomen or pelvis who received combinations of surgery, radiation, and chemotherapy vs surgery alone. JAMA Netw Open.

[12] Haciislamoglu E, Gungor G, Aydin G, Canyilmaz E, Guler OC, Zengin AY, Yenice KM (2020). Estimation of secondary cancer risk after radiotherapy in high-risk prostate cancer patients with pelvic irradiation. J Appl Clin Med Phys.

